# Wall Shear Stress Analysis and Optimization in Tissue Engineering TPMS Scaffolds

**DOI:** 10.3390/ma15207375

**Published:** 2022-10-21

**Authors:** Tiago H. V. Pires, John W. C. Dunlop, André P. G. Castro, Paulo R. Fernandes

**Affiliations:** 1 IDMEC, Instituto Superior Técnico, Universidade de Lisboa, 1049-001 Lisboa, Portugal; 2 MorphoPhysics Group, Department of the Chemistry and Physics of Materials, University of Salzburg, 5020 Salzburg, Austria; 3 ESTSetúbal, Instituto Politécnico de Setúbal, 2914-761 Setúbal, Portugal

**Keywords:** wall shear stress, computational fluid dynamics, optimization, bone tissue engineering, triply periodic minimum surfaces, simulated annealing

## Abstract

When designing scaffolds for bone tissue engineering (BTE), the wall shear stress (WSS), due to the fluid flow inside the scaffold, is an important factor to consider as it influences the cellular process involved in new tissue formation. The present work analyzed the average WSS in Schwartz diamond (SD) and gyroid (SG) scaffolds with different surface topologies and mesh elements using computational fluid dynamics (CFD) analysis. It was found that scaffold meshes with a smooth surface topology with tetrahedral elements had WSS levels 35% higher than the equivalent scaffold with a non-smooth surface topology with hexahedral elements. The present work also investigated the possibility of implementing the optimization algorithm simulated annealing to aid in the design of BTE scaffolds with a specific average WSS, with the outputs showing that the algorithm was able to reach WSS levels in the vicinity of 5 mPa (physiological range) within the established limit of 100 iterations. This proved the efficacy of combining CFD and optimization methods in the design of BTE scaffolds.

## 1. Introduction

In bone tissue engineering (BTE), scaffolds are porous support matrixes designed as an environment to promote cell proliferation, differentiation, and growth [1]. These cellular processes are considerably influenced by several parameters, one of which is the wall shear stress (WSS) that affects the cells inside the scaffold [2,3,4,5]. WSS is caused by the relative movement between the scaffold walls and the fluidic phase inside the scaffold. Studies have shown that different levels of WSS lead to different mechanical signals for the mesenchymal stromal cells, which, in turn, cause differences to the cellular differentiation process [6,7]. This parameter is affected by various factors, with one being the scaffold geometry. In fact, small changes in the scaffold’s design parameters (such as its porosity, surface topology, or curvature) considerably alter the average WSS experienced by the cells inside the scaffolds [8,9]. Furthermore, in order to promote the desired conditions to promote bone proliferation, the average WSS experienced by the cells needs to be between 0.1 and 10 mPa [10,11].

When designing scaffolds, the multiple possible inputs for scaffold development result in a large range of possible designs. Therefore, computational simulations are normally implemented to reduce the time and cost associated with conceptualizing a new scaffold. For BTE, computational fluid dynamics (CFD) analysis is essential in understanding how changes to a scaffold’s design influence its fluidic properties (such as the WSS) and the underlying cellular processes [12,13,14].

Additionally, computational simulations can also be used to design scaffold geometry with the desired characteristics, through a structural optimization approach. Although there are several different optimization methods, most of them follow the same framework [15]. After defining the design space, the chosen objective function, and the constraints for that specific optimization process, the process starts with an initial step where the initial geometry (initial solution) is set as well as the material properties of the structure. Afterward, the state variables are computed through a proper numerical tool in order to analyze the relevant properties of the initial geometry. If the resulting properties do not reach the objective, then an optimization algorithm is utilized to obtain a new geometry, according to the predefined constraints. This process repeats itself iteratively until a new structure satisfies the objective function. However, in BTE, optimization methods have rarely focused on the fluidic properties of the scaffold [14,15,16], instead only consider the scaffold’s mechanical properties (such as their Young’s modulus [17]; compressive strength [18] and octahedral shear strain [19,20,21]).

Taking this into consideration, the objective of this work was twofold. Firstly, the difference in average WSS was analyzed for different scaffold topologies, with either smoothed (tetrahedral elements) or non-smoothed (hexahedral elements) wall surface topologies. This was in order to determine whether previously designed numerical models [22] can be used in the study of WSS, or if an additional step to correct their surface topology is required. Secondly, a simulated annealing (SA) optimization strategy was used to design scaffolds for a specific average WSS, in order to investigate the feasibility of using structural optimization methodologies combined with CFD analysis in the design of scaffolds for required fluidic properties.

## 2. Materials and Methods

### 2.1. Scaffold Design

**Figure 1:**
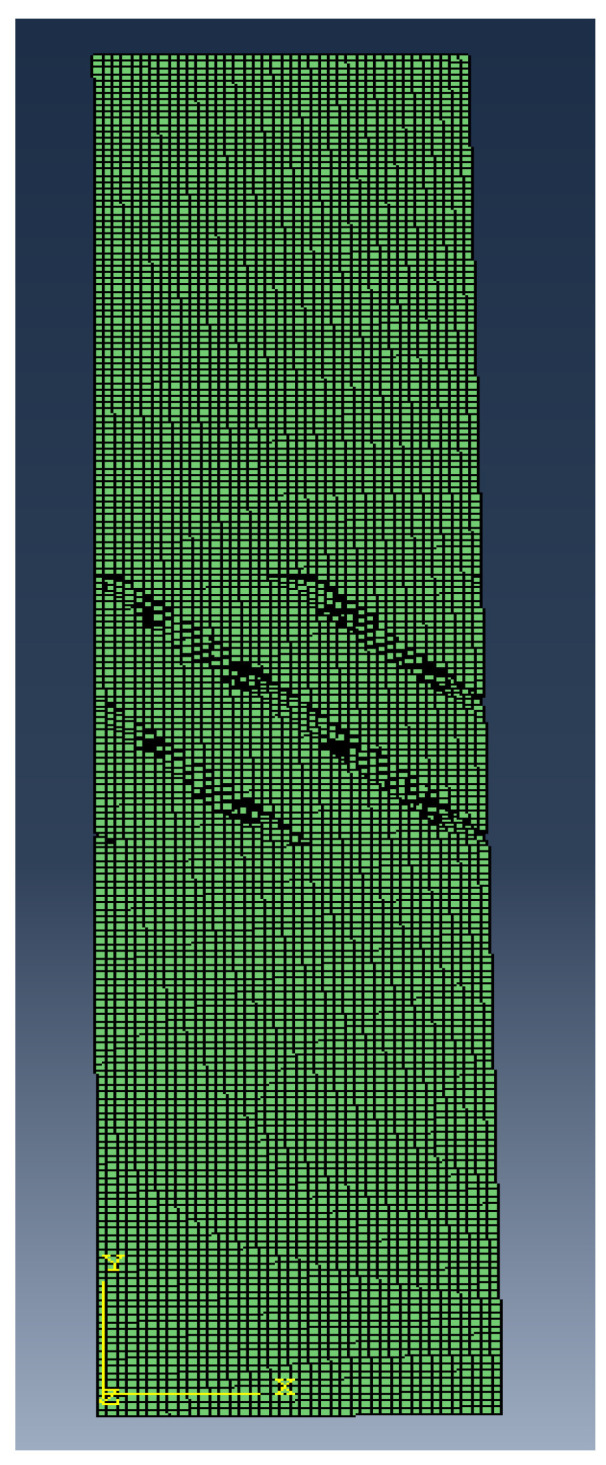
Example of a mesh with empty chambers before and after the scaffold.

To generate the scaffold’s mesh for the CFD analysis a computer code was used, which, given the specific design parameters, created a hexahedral mesh of the desired geometry for the analysis [22]. This mesh consists of a single cubic unit of the fluidic phase of the chosen scaffold design, with forty elements per side, seeing as previous studies have shown that 40 elements per side are the minimum in order to obtain an appropriate scaffold geometry [23]. For this study, the chosen scaffold geometries were the triply periodic minimum surfaces (TPMS) gyroid (SG) and Schwartz diamond (SD) designs. This choice was because these scaffolds were found to be the most appropriate TPMS structures for cellular growth given their high permeability and fluid tortuosity that promotes cell–scaffold interaction [22]. Additionally, an empty portion was added before and after the scaffold, which represented the empty permeability chamber that allows the fluid flow to stabilize before and after passing the scaffold (Figure 1), mimicking the experimental setup for permeability analysis [24]. 

**Figure 2:**
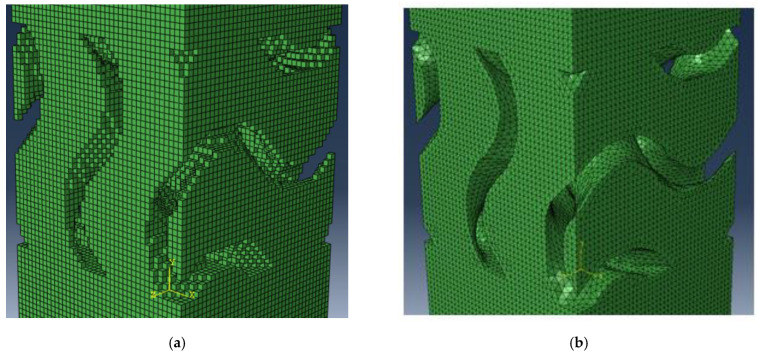
Example meshes: (**a**) original hexahedral scaffold mesh; (**b**) smoothed hexahedral scaffold mesh.

Having designed a scaffold mesh with hexahedral elements (Figure 2a), to create the corresponding mesh with tetrahedral elements and a smooth surface, an additional design step was required. To this end, Meshlab^®^ [25] was used to apply a three-step Laplacian smoothing process followed by a three-step isotropic explicit remeshing process to obtain the scaffold with smooth surfaces (Figure 2b).

The scaffold design parameters considered for this paper were: the porosity of the scaffold and the length of the side of a single basic cubic unit (higher values result in scaffolds with larger pore sizes). In addition to these two characteristics, two other parameters that were analyzed were the length of empty chambers before and after the scaffold and, in the case of the tetrahedral meshes, the world unit parameter from Meshlab’s isotropic explicit remeshing process (which directly controls the number of elements in the final mesh). Given that these factors were constant across all scaffolds that were studied, a convergence test was carried out for each one to determine their best value. For these tests, both scaffold geometries were analyzed in regard to their average WSS and permeability with a fluid inlet velocity of 0.0001 m/s, a length of 5 mm for the cubic unit, and a 70% porosity.

### 2.2. Surface Topology CFD Analysis

Having defined the optimal values for the cubic unit length and the world unit parameters, the next step was to define the scaffold designs to be compared. For each of the two TPMS geometries, three different levels of porosity were considered (60%, 70%, and 80%) for a total of six different scaffold designs. A hexahedral and a tetrahedral mesh were then created for each design and their average WSS was studied using CFD analysis with the previously mentioned fluid inlet velocity of 0.0001 m/s and cubic unit length of 5 mm. For this study, the computational simulations were conducted using the commercial software Fluent^®^ Ansys^®^ (Ansys Inc., Canonsburg, PA, USA), which has already proven to be a useful tool for analyzing the fluidic properties of scaffolds [26,27]. In terms of the parameters for the Fluent solver, the fluid chosen for the simulations was water, in accordance with previous studies [22,28], with a density of 1000 kg/m^3^ and dynamic viscosity of 0.001 Pa.s. The fluid was also assumed to pass through the scaffold in a steady state laminar flow.

Three different boundary conditions need to be defined, these being the velocity inlet, the pressure outlet, and the wall boundary. For these simulations, it was considered that the fluid was traveling in the y direction through the scaffold. This meant that an exterior wall on the xz plane was the velocity inlet surface, while the opposite wall was the pressure outlet with 0 Pa, so that the pressure drop of the scaffold would be equal to the pressure at the inlet (since the pressure drop is the pressure at the inlet minus the pressure at the outlet). Additionally, interfaces with periodic boundary conditions were implemented in parallel on the remaining exterior walls in order to create an infinite scaffold in the x and z directions.

Finally, after running the simulations, the software CFD-post from Ansys was used to calculate the average WSS on the scaffold walls and, if needed, the pressure difference between the inlet and outlet of each scaffold. Afterward, the scaffold permeability could be calculated using the pressure drop and Darcy’s law (Equation (1)) [29,30]:

K = (v·µ·L)/ΔP,(1)

where K is the permeability expressed in m^2^; ΔP is the pressure drop expressed in Pa; L is the length of the scaffold expressed in m; v is the inlet velocity of the fluid and µ is the dynamic viscosity of the water which is 0.001 Pa*s. 

### 2.3. Structural Optimization Process

For this work, the created optimization algorithm was focused on the scaffold’s average WSS on both the SD and SG geometries with tetrahedral elements. More specifically, the algorithm will attempt to design a scaffold geometry with an average WSS that promotes cellular proliferation and growth. Zhao et al. (2015) [10] and Ali et al. (2019) [11] discussed how the WSS of structures meant for bone growth needed to be between 0.1 and 10 mPa. Given that the present method looks at the average WSS, a target was set at 5 mPa for the optimization program, as an average target value between the two edges of the accepted physiological levels. Additionally, seeing as the average WSS of the scaffold was dependent on the fluid velocity passing through it (unlike permeability, which is only dependent on the scaffold design), a constant inlet velocity of 0.001 m/s (similar to the parameter described in a comparable scaffold perfusion analysis [31]), was selected to allow a comparison between all scaffold geometries.

To solve the established scaffold optimization challenge, the metaheuristic optimization computational algorithm simulated annealing (SA) was chosen. This is a metaheuristic optimization approach used to solve optimization problems with a large search space. Additionally, this algorithm allows a worse solution to be accepted in the earlier iterations, which minimized the probability of the program reaching a local minimum and being unable to progress further. 

SA requires the definition of an appropriate cooling schedule variable (a); the objective function; the maximum number of iterations the algorithm runs before stopping (K_max_); and the upper (U_j_) and lower (L_j_) boundaries of each parameter. In this work, a cooling schedule variable of 0.9 was chosen, alongside a maximum of 100 iterations. The objective function was defined as the normalized difference between the target (5 mPa) and the real average calculated WSS, and the design variables are the side length of the unit cell of the TPMS structure and the scaffold porosity. These variables are limited to the lower and upper bounds of 1 and 10 mm for the unit length and 60% and 80% for the porosity, respectively. These values were chosen to maintain pore sizes that promote cellular processes. 

After defining the variables, the algorithm then calculated an initial value for each parameter (equal to the halfway point between the upper and lower boundary of each parameter), and used the process discussed in the previous section to create and study the average WSS of the scaffold. 

The program then used a Matlab^®^ (Mathworks, Natick, MA, USA) script to calculate the objective function value of the designed scaffold and recorded it as the current solution (Z_c_). Subsequently, the algorithm defined an initial temperature (T_j_) equal to 1/5 of the current solution. The temperature was cooled after each iteration by multiplying it by the previously established cooling schedule variable (a).

Having the initialization of the SA algorithm, the iterative process started with testing the immediate neighbors of the current solution (using the current parameters and normal distribution). If a parameter went outside the predefined boundaries, then it was replaced by the closest values within the boundaries (for example, if the new porosity parameter was equal to 85%, then the code replaced it with 8’, the corresponding maximum allowed value). The program then calculated the objective function value of the new parameters, using the same method as discussed above, and recorded it as the next trial solution (Z_n_). If Z_n_ was lower than or equal to Z_c_, then the code always accepted the new value. Otherwise, the chance of accepting the new value was equal to $e^{{((Z}_{c}{-Z}_{n}{)/T}_{j})}$. The code then recorded the value of the parameters and objective function value and proceeded with the temperature cooling. Finally, when the algorithm ended, it returned the history of the optimization process.

## 3. Results

### 3.1. Scaffold Design Parameters

**Figure 3:**
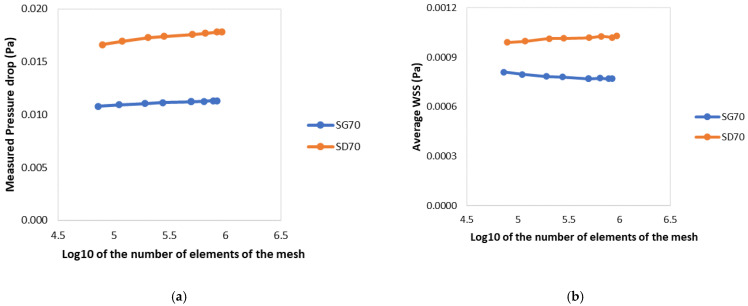
Comparison between different values of the world unit parameter, resulting in tetrahedral meshes with varying element numbers, regarding the test scaffold’s: (**a**) pressure drop; (**b**) average WSS.

Figure 3 shows the results of the convergence analysis on the pressure drops and average WSS for simulations conducted on both 70% porosity test scaffolds. After obtaining these results, the relative difference between the analysis with the smallest world unit and every other simulated mesh was calculated (Table 1 and Table 2). As expected, the results showed that an increase in the number of elements in the simulation led to more accurate results. However, an increase in the number of elements also resulted in longer simulations. Therefore, a compromise had to be made between the accuracy of the results and the computational cost of the simulation. Accordingly, the world unit value 0.15 was decided as the best option seeing as it always presented a considerably low relative error (almost always around or below 1%) with considerably quicker simulations than models with a higher element count.

For the fluid flow to stabilize, the chambers before and after the scaffold must have sufficient “empty” length to allow the flow to stabilize before and after the scaffold; otherwise, this could affect the numerical simulation, namely the pressure drop measurement. However, the longer the empty chamber, the higher the computational cost of the simulations, which would in turn considerably affect the computational cost of the optimization algorithm. Therefore, the desirable empty chamber length is the shortest one that still allows the stabilization of the fluid flow. Furthermore, an additional length should be added to the minimum length to account for different scaffold configurations that require a longer chamber to stabilize, such as lower porosity scaffolds. 

In order to determine a minimal length, two different scaffolds were designed: one where the length of the empty chambers, before and after the scaffold, was equal to the length of the basic cubic unit and another where the length empty chambers was equal to half the length of the basic cubic unit. As the fluid flow pressure drop measurement and the average scaffold WSS are both independent from the length of the chambers (as long as the flow is stable at both ends of the numerical model), if both CFD simulations returned similar pressure drop and average WSS values, then this meant that the model with the smaller empty chamber was enough to allow the fluid flow to stabilize. 

**Figure 4:**
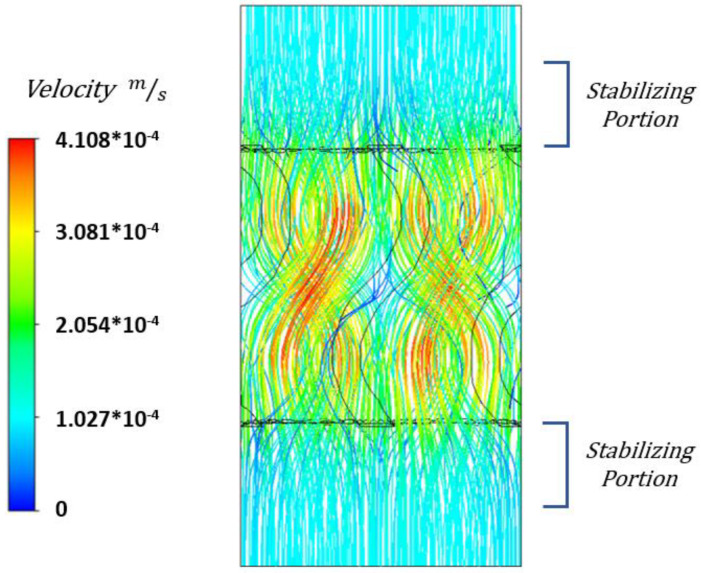
Fluid flow streamlines of a 1 mm sided SG scaffold with 70% porosity.

As shown in Table 3, for both the SG and SD scaffolds, the difference between the models with varying values of h was minimal, with their difference always below 0.3%. This indicates how the shorter chamber was sufficient in allowing the flow to stabilize at the edges of the numerical model. On top of that, analyzing the fluid flow streamlines presented in Figure 4 highlights how the fluid flow is completely stable at the edges of the numerical model. However, choosing a shorter chamber could potentially risk the CFD simulations for the scaffolds with the smallest pore sizes whilst not significantly contributing to a lower computational cost. Therefore, an empty chamber length of half the length of the scaffold was chosen for all the CFD analysis. 

### 3.2. Surface Topology CFD Analysis

Table 4 and Table 5 illustrate how the difference in the of scaffold surface topology (original jagged surfaces with hexahedral elements or smooth surfaces with tetrahedral) influences the average WSS of the six different scaffold designs. The results show that all of the smoothed scaffolds had a higher average WSS than the original scaffolds (average of 35% increase).

Additionally, the volumes of the original and smoothed scaffolds were compared to determine if the smoothing process would influence the porosity of the scaffolds (Table 6).

### 3.3. Optimization Method

**Figure 5:**
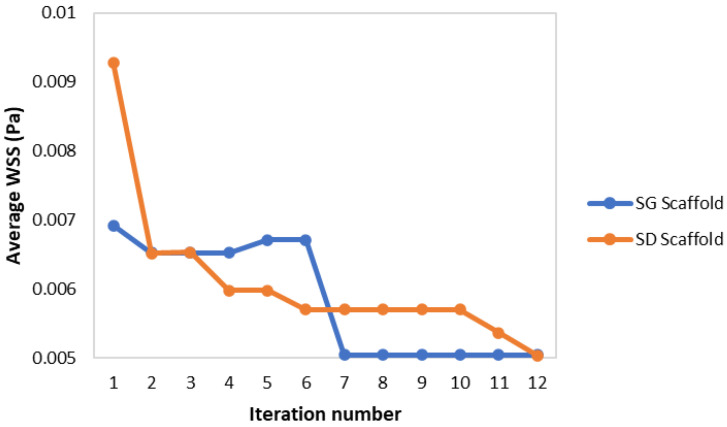
Convergence of the average WSS for the SA algorithm on the SD and SG scaffolds.

For each scaffold geometry, the optimization algorithm ran multiple times. Examples of one optimization run for the SD and SG geometries are shown in Table 7 and Table 8, respectively. The iterations where no new point was considered were not presented in the tables. The convergence of the results is shown in Figure 5.

## 4. Discussion

The comparison between the different surface topologies showed that the WSS levels in the smoothed surfaces increased by an average of 35% when compared to the original non-smoothed surfaces. A previous study has shown that in scaffolds with equal geometry but different surface roughness levels, a lower average WSS was calculated for the scaffolds with a higher roughness [32]. This corresponds to what was observed in Table 5, where the original jagged mesh, given its higher surface roughness, resulted in a lower WSS than the smoothed tetrahedral mesh. Furthermore, when fabricating scaffolds using 3D printing techniques, these methods normally create surface topologies closer to the smoothed surfaces than the original jagged ones. Therefore, the smoothed surface was chosen as the basis for the optimization algorithm of the scaffold’s average WSS. Finally, the difference between the volume before and after the smoothing is illustrated in Table 6. The difference between the two is minimal, indicating that the smoothing process can be used without it affecting the scaffold’s porosity.

In terms of the optimization algorithm, it was able to consistently reach the desired average WSS of 5 mPa taking a maximum of 12 iterations, well within the established 100-iterations limit. However, given the inherent random nature of the SA algorithm, the number of iterations and the final parameters varied between each optimization run. Nevertheless, the outputs remained similar to those reported in Table 7 and Table 8. The optimal SD scaffold was always close to the maximum limit of 10 mm for the cubic unit length and a porosity close to 70%; whilst the SG scaffolds had a cubic unit length between 7 and 8.5 mm and porosity between 63% and 73% (higher porosity corresponded with lower cubic unit length).

The resulting scaffold designs from this algorithm were able to refine the scaffold geometry in order to reach the desired average WSS of 5 mPa. This highlights how the optimization algorithm presented in this work can be combined with CFD analysis; additionally, this algorithm can enhance the design of TPMS scaffolds with a specific fluidic property such as a specific average WSS or permeability. The results also demonstrate how the SA method worked as intended, with the code accepting worse solution to the current one at earlier iterations (for example the SG optimization from the second to the fifth iteration as seen in Table 8) and only accepting strictly better solutions at higher iterations (when the temperature variable T_j_ of the algorithm was at its lowest).

Although the SA algorithm proved as useful to optimize and improve one specific parameter, it would not be able to simultaneously improve multiple fluidic parameters, such as both WSS and permeability. In other words, WSS and permeability are inversely correlated, with studies have shown that that increasing the average WSS leads to a decrease in the scaffold’s permeability and vice versa [33,34]. To address the limitations of the current algorithm, a multi-objective optimization approach might be implemented in future developments. Instead of focusing on a single objective function, multi-objective optimization could analyze each parameter individually and would give a set of possible solutions, organized as a Pareto front, but with a higher computational cost. This method would result in multiple possible solutions, which would need to be analyzed in terms of their suitability for BTE.

## Figures and Tables

**Table 1:** Calculated pressure drop and average WSS for the SD70 scaffold.

**World Unit**	0.095	0.1	0.125	0.15	0.175	0.2	0.25	0.3
**# Elements**	936246	840421	662816	503793	282630	203490	118248	78767
**Pressure Drop (mPa)**	17.846	17.817	17.713	17.587	17.401	17.283	16.937	16.635
**WSS (mPa)**	1.029	1.021	1.028	1.018	1.016	1.013	0.998	0.990

**Table 2:** Calculated pressure drop and average WSS for the SG70 scaffold.

**World Unit**	0.095	0.1	0.125	0.15	0.175	0.2	0.25	0.3
**# Elements**	844429	777951	645069	499543	275998	190338	110911	72858
**Pressure Drop (mPa)**	11.319	11.305	11.260	11.210	11.131	11.061	10.922	10.790
**WSS (mPa)**	0.770	0.770	0.774	0.768	0.781	0.785	0.797	0.811

**Table 3:** Comparison of the pressure drop and average WSS between scaffolds with different length of the empty chamber before and after the scaffold.

	SG70 Half Chamber	SG70 Full Chamber	SD70 Half Chamber	SD70 Full Chamber
Pressure Drop (mPa)	11.212	11.187	17.587	17.555
Relative Difference (%)	−0.224		−0.172	
Average WSS (mPa)	0.768	0.768	1.018	1.021
Relative Difference (%)	−0.028		0.287	

**Table 4:** Comparison of the average WSS between SG scaffolds with and without surface topology smoothing.

	SG60 Original	SG60 Smoothed	SG70 Original	SG70 Smoothed	SG80 Original	SG80 Smoothed
Average WSS (mPa)	0.700	0.930	0.568	0.768	0.455	0.625
Relative Difference (%)	32.843		35.221		37.320	

**Table 5:** Comparison of the average WSS between SD scaffolds with and without surface topology smoothing.

	SD60 Original	SD60 Smoothed	SD70 Original	SD70 Smoothed	SD80 Original	SD80 Smoothed
Average WSS (mPa)	0.955	1.278	0.749	1.018	0.607	0.821
Relative Difference (%)	33.826		35.915		35.180	

**Table 6:** Volume (mm^2^) of various scaffolds with square size of 1 mm before and after smoothing.

	G60	G70	G80	SD60	SD70	SD80
Original	599.5	700.5	799.8	600.8	699.1	800.5
Smoothed	599.4	701.4	801.6	599.1	699.2	801.71
Relative Difference (%)	−0.012	0.128	0.216	−0.280	0.026	0.155

**Table 7:** Simulated annealing process for the SD scaffolds.

Optimization Iteration	Cubic Unit Length (mm)	Porosity (%)	Average WSS (mPa)
1	5.500	70	9.277
2	7.375	73.1	6.515
3	7.735	70.8	6.531
4	9.523	65.4	5.978
6	10.000	65.4	5.697
11	10.000	68.2	5.361
12	10.000	71.2	5.039

**Table 8:** Simulated annealing process for the SG scaffolds.

Optimization Iteration	Cubic Unit Length (mm)	Porosity (%)	Average WSS (mPa)
1	5.500	70	6.916
2	6.183	67.2	6.522
5	6.081	66.5	6.710
7	7.320	71.6	5.041

## Data Availability

The generated models can be made available upon reasonable request to the corresponding author.
